# *Pa*REx: an open-source pipeline for the automated analysis of *Pseudomonas aeruginosa* resistomes from whole-genome sequences

**DOI:** 10.1128/aac.01326-25

**Published:** 2026-03-16

**Authors:** Carla López-Causapé, Matias Bonet, Biel Taltavull, Paola Medina-Retiga, Miquel A. Sastre-Femenía, Sara Cortés-Lara, María A. Gomis-Font, Fernando Gomez-Romano, Antonio Oliver

**Affiliations:** 1 Servicio de Microbiología, Hospital Universitario Son Espases, Instituto de Investigación Sanitaria Illes Balears 375118, Palma de Mallorca, Spain; 2 CIBER de Enfermedades Infecciosas (CIBERINFEC), Instituto de Salud Carlos III 38176, Madrid, Spain; 3 OceanDrivers, Palma de Mallorca, Spain; University of Fribourg, Fribourg, Switzerland

**Keywords:** bioinformatics, whole-genome sequencing, resistome, *Pseudomonas aeruginosa*

## Abstract

Whole-genome sequencing (WGS) has become a valuable tool for bacterial typing and resistome analysis. However, most existing antibiotic resistance databases and bioinformatics tools are not useful for *P. aeruginosa* resistome profiling as they fail to detect mutation-driven antibiotic resistance mechanisms. In addition, the increasing diversity of AmpC variants (*Pseudomonas-*derived cephalosporinases [PDCs]) in recent years adds complexity to *P. aeruginosa* resistome profiling. To address these challenges, we have developed *Pa*REx, an open-source automated pipeline specifically designed for the analysis of *P. aeruginosa* mutation-driven (221 chromosomal genes) and horizontally acquired resistome from whole-genome sequences, along with the PDC analyzer web tool, a user-friendly platform that designates PDCs and detects resistance to novel antipseudomonal β-lactams. The utility of *Pseudomonas aeruginosa* Resistome Explorer (*Pa*REx) was demonstrated through the analysis of 260 *P. aeruginosa* isolates, using its output to calculate five previously defined genotypic resistance scores, achieving results comparable to that of manual bioinformatic analysis. Likewise, the PDC analyzer web tool was validated with more than 30,000 global *P. aeruginosa* genomes, demonstrating its utility for PDC designation and detection of mutations conferring resistance to novel BL/BLI. Both *Pa*REx and the PDC analyzer web tool were shown to be user-friendly, accurate, and automated tools for resistome profiling in *P. aeruginosa*, enhancing current capabilities of existing tools. However, an ongoing refinement of these tools and its custom-built databases is needed to address remaining gaps and future challenges of whole-genome sequence-based resistance prediction.

## INTRODUCTION

*Pseudomonas aeruginosa* is one of the most frequent and severe causes of nosocomial infections, being the first cause of ventilator-associated pneumonia and particularly affecting intensive care units and immunocompromised patients (1). Likewise, *P. aeruginosa* is the most frequent driver of chronic respiratory infections in individuals with cystic fibrosis or patients with other chronic pulmonary conditions (2, 3).

Moreover, in recent years, the global prevalence of infections caused by multidrug-resistant (MDR) and extensively drug-resistant (XDR) *P. aeruginosa* strains has increased worldwide, significantly compromising and limiting the selection of effective antimicrobial therapies (1). This global threat results from a complex interplay in which the extraordinary ability of *P. aeruginosa* to develop resistance to nearly all available antimicrobials through the acquisition of chromosomal mutations, along with an increasing prevalence of horizontally acquired resistance determinants in this pathogen, and the nosocomial spread of certain *P. aeruginosa* high-risk clones worldwide play a major role (4).

Despite novel antipseudomonal agents being recently introduced into clinical practice contributing to mitigate this problem, carbapenem-resistant *P. aeruginosa* remains a major concern for public health and has been classified as a high-priority pathogen in the World Health Organization’s Bacterial Pathogen Priority lists (5). In this context, the development of robust tools for accurate characterization of *P. aeruginosa* resistomes would be very helpful for the design of therapeutic strategies, for monitoring and preventing resistance development, and for establishing effective infection control measures.

As high-throughput sequencing technologies have become increasingly affordable, many clinical and research microbiology laboratories have introduced their use in routine practice, making both bacterial typing and characterization of bacterial resistomes possible in a single assay. However, the analysis of whole-genome sequences (WGS) requires advanced bioinformatics skills and the use of different open-source software, databases, and tools. To facilitate these analyses, bioinformatic pipelines, such as BacPipe or Bactopia that integrates different parts of the analysis (6, 7), or even web platforms such as the Center for Genomic Epidemiology (https://genepi.dk/), which provides user-friendly tools such as ResFinder (8), have been developed in recent years. However, these pipelines and platforms are not pathogen-specific and use generic antibiotic resistance databases such as the ResFinder (9, 10), the Comprehensive Antibiotic Resistance Database CARD (11), or the NCBI AMRFinderPlus (12) databases, limiting resistome characterization of bacterial isolates to the detection of acquired resistance genes and some specific chromosomal resistance mutations. Unfortunately, these tools are not useful for the characterization of *P. aeruginosa* resistomes as in this pathogen, mutation-driven mechanisms play a major role in antibiotic resistance. Thus, the investigation of *P. aeruginosa* resistomes from whole-genome sequencing data requires highly time-consuming manual analysis and expertise on *P. aeruginosa* resistance mechanisms and genomics (13). Moreover, in recent years, and largely due to the introduction of novel beta-lactam/beta-lactamase inhibitor combinations into the clinical practice, there is a growing diversity and emerging role in resistance of AmpC variants (known as *Pseudomonas*-derived cephalosporinases [PDCs]) (14). Thus, an automated pipeline specifically designed for establishing *P. aeruginosa* antibiotic resistance genotypes, including PDC designation, is needed.

In this work, we introduce *Pseudomonas aeruginosa* Resistome Explorer (*Pa*REx), an open-source pipeline for the automated analysis of *P. aeruginosa* resistomes from whole-genome sequences, which benefits from the use of different open-source and public bioinformatics tools, software, and databases along with custom-built databases and tools, enabling an accurate resistance genotype profiling of *P. aeruginosa* isolates.

## RESULTS AND DISCUSSION

### *Pa*REx design and implementation

**Fig 1:**
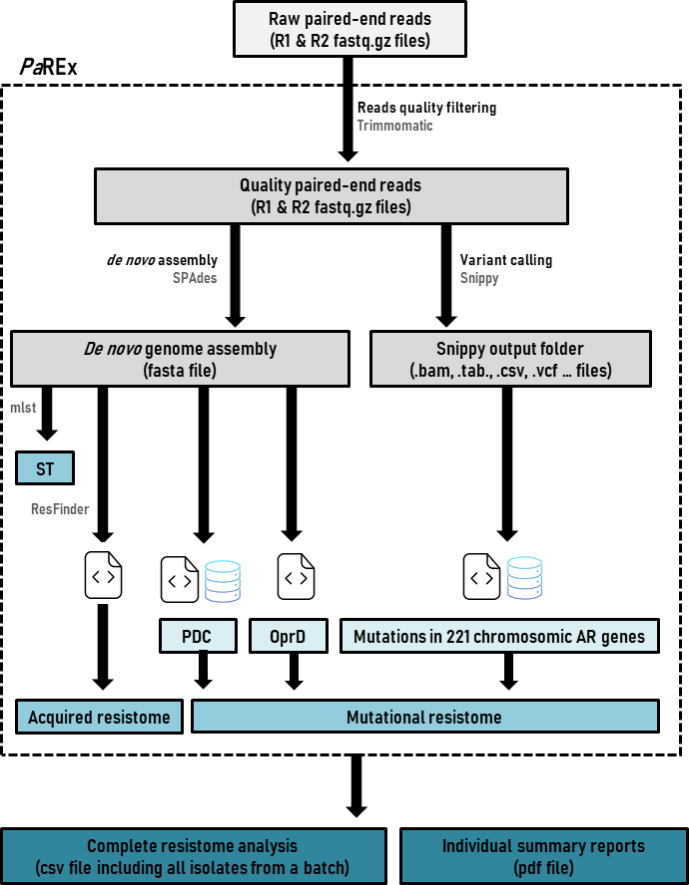
Overview of the *Pa*REx pipeline.

The *Pa*REx is an open-source Python-based customizable pipeline that has been specifically designed for the automated analysis of *P. aeruginosa* resistomes from Illumina paired-end reads. *Pa*REx uses different open-source bioinformatics tools, software, and publicly available databases along with custom-built databases, scripts, and tools and is composed of two main components: the *Pa*REx pipeline and the *Pa*REx databases. An overview of *Pa*REx is represented in Fig. 1.

As shown, *Pa*REx comprises different steps that have been further detailed in the Methods and Materials section. Third-party open-source tools used by the *Pa*REx pipeline are listed in Table 1 with their individual version numbers.

**TABLE 1:** List of third-party tools and databases used by *Pa*REx

Name and version	Description
Trimmomatic v0.39	A flexible read trimming tool for Illumina reads
SPAdes v3.15.3	A *de novo* genome assembler for Illumina reads
Snippy	A tool for rapid haploid variant calling and core genome alignment
mlst	A tool to scan contig files against PubMLST typing schemes
ResFinder	A tool (v4.5.0) and database (v2.1.0) for the identification of acquired antimicrobial resistance genes

### Validation of *Pa*REx functionalities

A total of 260 *P. aeruginosa* clinical isolates were analyzed for the validation of *Pa*REx functionalities using the batch mode. The csv summary file containing the compiled results for all the isolates and some of the individual pdf summary reports have been included in the Data S1 and S2 as examples of *Pa*REx’ final reports.

The analysis of the 260 isolates took about 49 h with 8 CPUs, processing 188.7 GB of compressed input data (854.2 GB uncompressed) and generating a total output of 1.5 TB. Table 2 summarizes the detailed run times and output size for each step of the *Pa*REx pipeline.

**TABLE 2:** Run times and outputs generated in each *Pa*REx step

*Pa*REx’ step	Operation name	Running time	Output
Decompress fastq.gz files	Unzip	2 h 35 min	854.2 GB
Read quality filtering	Trimmomatic	6 h 17 min	567.2 GB
*De novo* assembly	SPAdes	1d 1h 13 min	1.8 GB
Multilocus sequence typing (MLST), resistome characterization, and summary reports generation	Resistome	15 h 6 min	112.7 GB

*Pa*REx was able to assign a sequence type (ST) to 92% (239/260) of the isolates. Those for which an ST was not assigned harbored a new MLST allele (*n* = 11) that was not included in the database, represented a new combination of the MLST alleles (*n* = 3), or contained some MLST allele that was not completely covered (*n* = 7). Within the whole collection, 151 different STs were detected, being ST175 (*n* = 14; 10 XDR, 1 MDR, and 3 modR), ST244 (*n* = 11; 1 MDR, 3 modR, and 7 S), and ST253 (*n* = 10; 1 XDR, 1 MDR, 1 modR, and 7 S), detected in 10 or more isolates.

*P. aeruginosa* XDR ST175 is a high-risk clone widely distributed in Spain and is characterized by several genetic resistance markers, including the acquisition of the aminoglycoside-modifying enzyme *ant(2’’)-Ia* (*aadB*) and the presence of specific resistance mutations in its chromosomal genes *gyrA* (T83I and D87N)*, parC* (S87W and L168Q), and *mexZ* (G195E) (15–17). *Pa*REx reported the presence of the *ant(2’’)-Ia* aminoglycoside-modifying enzyme (100% identity) and *parC* mutations in all 10 XDR ST175 isolates as well as the characteristic mutations in the quinolone resistance-determining region of GyrA (T83I: 10/10 and D87N: 9/10) and MexZ (9/10) in almost all. Other resistance mutations previously reported among XDR ST175 Spanish isolates, such as the Q142X-inactivating mutation in the OprD porin and the G154R gain-of-function mutation in the *ampC* cephalosporinase regulator AmpR, were detected in 2 of them (15–17). Moreover, in 2 XDR ST175 isolates, *Pa*REx reported the presence of *bla*_VIM-2_ (*n* = 2) along with either a *bla*_OXA-2_ or a *bla*_OXA-210_ gene, determinants also previously described in specific lineages of this high-risk clone (17).

Among the other 11 XDR isolates included in the collection used for *Pa*REx validation, 4 belong to high-risk clone ST235 and 1 to high-risk clone ST111, these clones being characterized by the frequent horizontal acquisition of resistance gene determinants (4, 18). Accordingly, in all XDR ST235 and ST111 isolates, *Pa*REx reported the presence of a horizontally acquired beta-lactamase along with aminoglycoside-acquired resistance genes.

Of note, for isolates exhibiting a susceptible phenotype (*n* = 165), *Pa*REx did not detect any acquired beta-lactamase gene and any acquired aminoglycoside resistance genes. Likewise, on average, *Pa*REx reported a minor number of resistance mutations within the basic resistome chromosomal genes for these isolates compared to those exhibiting multidrug-resistant and extensively drug-resistant phenotypes: 2.3 (S) vs 5.3 (MDR) and 7.7 (XDR).

Moreover, in previous studies, we developed and validated a genotypic scoring system based on the manual analysis of acquired resistance determinants and mutations within 40 chromosomal genes from whole-genome sequences with the aim to predict *P. aeruginosa* phenotypic susceptibility to ceftazidime (CAZ), ceftolozane/tazobactam (TOL/TZ), meropenem (MER), ciprofloxacin (CIP), and tobramycin (TOB) (13, 19, 20). Score values under 0.5 points were intended to predict phenotypic susceptibility, specifically, susceptibility at increased exposure for CAZ and CIP, and susceptibility at standard dosing for TOB, TOL/TZ, and MER. Conversely, score values equal to or above 1 were intended to predict a resistance phenotype, whereas intermediate score values (0.5 to <1) were considered indeterminate, with the exception of MER, for which these values should predict susceptibility at increased exposure. As manual resistome analysis in *P. aeruginosa* is very time-consuming and requires expertise on *P. aeruginosa* resistance mechanisms and genomics, its automatization can significantly reduce the turn-around time of the scoring system. Therefore, to demonstrate the potential utility of *Pa*REx for this purpose, the *Pa*REx final report was used for calculating the genotypic scores (Data S3). Table 3 summarizes the categorical agreement (CA) and the minor (mE), major (ME), and very major (VME) errors rates obtained for each antibiotic.

**TABLE 3:** Performance of the genotypic scoring system using the *Pa*REx output

Antibiotic	Indeterminate score values (%)	CA (%)	mE(%)	ME (%)	VME (%)
CAZ	13.8	91.2	–^*a*^	4.4	4.4
TOL/TZ	7.3	98.3	–	0	1.7
MER	–	75.0	21.1	2.7	1.1
CIP	10.4	91.8	–	1.3	6.9
TOB	10.4	98.7	–	0.9	0.4

^*a*^ –, not applicable.

As shown, the major error rate for ceftazidime was >3%, and very major errors rates for ceftazidime, ceftolozane/tazobactam, and ciprofloxacin were >1.5% and thus would fall out of the limits established by ISO 20776-2:2021 (21). These numbers are, however, quite similar to those extracted from the original study reporting the scoring system (CAZ: indeterminate 20.6%, CA 93.1%, ME 5%, and VME 1.9%; TOL/TZ: indeterminate 9.3% and CA 100%; CIP: indeterminate 11.3%, CA 95%, ME 2.8%, and VME 2.2%) (13) and thus confirm the utility of *Pa*REx for the automatic analysis of *P*. *aeruginosa* resistomes. Indeed, genotype-phenotype discrepancies might be attributed to different contributing factors. First, since validation of the genotypic scores was not the primary aim of this work, there exists a potential risk that discrepancies may result from experimental errors as minimum inhibitory concentrations (MICs) were determined only once per isolate as well as whole-genome sequencing. In addition, some of the observed discrepancies may be linked to limitations in the *Pa*REx approach, as, for instance, the current version does not investigate the presence of large deletions that can impact phenotypic resistance. Moreover, in *Pa*REx, the detection of resistance mutations relies on a variant calling analysis, and the inclusion of bioinformatics analysis based on the *de novo* assemblies could perhaps increase the detection of resistance mutations in some instances. Moreover, encountered sequence variants are filtered using a large list of natural polymorphisms, but since it may still not be fully saturated, variants not linked to antibiotic resistance could still be present. Finally, significant gaps remain in the understanding of *P. aeruginosa* resistance mechanisms and its complex interplay. Thus, further and continuous refinement of the genotypic score system is needed to improve its predictive accuracy. Despite these discrepancies, the *Pa*REx pipeline constitutes a significant advancement by considerably reducing times and the expertise required for the analysis of *P. aeruginosa* resistomes from whole-genome sequences that can eventually be applied for this or other purposes such as resistance surveillance.

### The PDC analyzer web tool

In addition to *Pa*REx, we have developed the PDC analyzer web tool, which is available at: https://arpbigidisba.com. This is a user-friendly web tool based on the PDC designation module included in the *Pa*REx pipeline. In addition, to inform users about the detected PDC allele variant and the amino acid variations encountered relative to the PAO1 reference cephalosporinase sequence (PDC-1), the PDC analyzer web tool additionally provides a warning message if a mutation associated with resistance and/or that may contribute to resistance to novel antipseudomonal cephalosporins, such as ceftolozane-tazobactam, ceftazidime-avibactam, and/or cefiderocol, is detected (Data S4). Conversely, the PDC analyzer web tool also alerts when a deleted (<90% coverage) or nonfunctional PDC is detected (premature stop codons or frameshift mutations).

### Validation of the PDC analyzer web tool

Mack and collaborators (22) recently explored the presence of β-lactamase alleles in a global collection of 30,452 *P. aeruginosa* genomes, encountering that *bla*PDC was the most common *bla* gene family (30,842 genes). In that work, a known PDC allele variant was assigned in 29,369 isolates (254 distinct alleles), whereas in 1,144, an unknown PDC allele variant was detected (622 distinct alleles). To validate the PDC analyzer web tool and to determine the global prevalence of mutations linked to resistance to the novel antipseudomonal combinations ceftolozane/tazobactam, ceftazidime/avibactam, and cefiderocol in *P. aeruginosa,* we analyzed the 863 distinct PDC allele variants for which an accession number was available at the National Center for Biotechnology Information database.

The PDC analyzer web tool reported 593 alleles as new types (68.7%). Among the 270 alleles included in the PDC analyzer database, 67 alleles with mutations associated with resistance and 21 alleles with mutations that may contribute to resistance against novel antipseudomonal cephalosporins were detected. Likewise, 77 alleles with mutations associated with resistance and 39 alleles with mutations that may contribute to resistance were detected among the new allele types. Of note, in 14 PDC alleles, more than 1 resistance mutation was detected.

**Fig 2:**
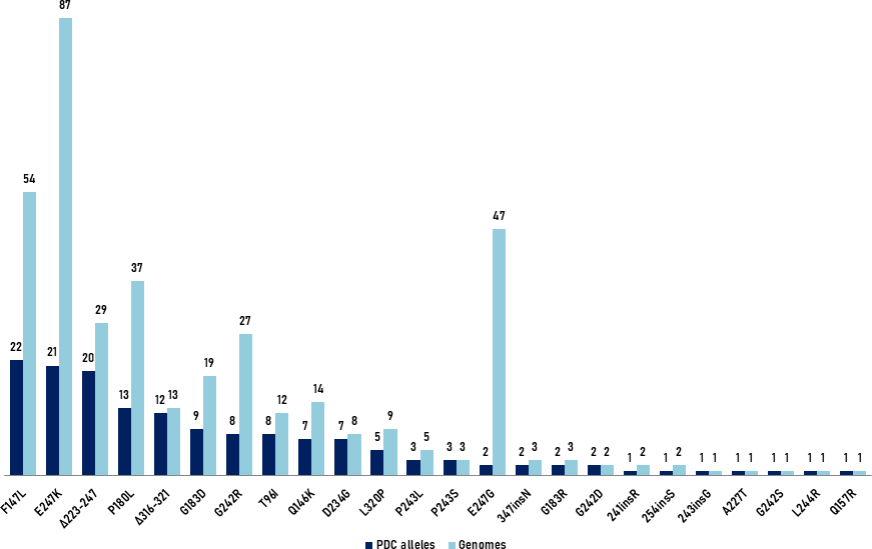
PDC alleles and total genome counts per resistance mutation detected with the PDC analyzer web tool.

For each mutation associated with resistance identified with the PDC analyzer web tool, the corresponding counts of PDC alleles and genomes carrying each mutation are represented in Fig. 2.

The PDC analyzer tool identified 24 different resistance mutations, with E247K/G, F147L, and P180L substitutions being the most frequently detected, along with mutations affecting the omega- (223–247) and the R2-loops (316–321). Of note, although the E247G mutation was present in 47 isolates, it was only associated with 2 different PDC alleles, suggesting that it may be related to the clonal dissemination of some specific ST.

In addition, 2 mutations that may contribute to resistance were detected: V239A and R126H; of note, V239A was identified in 57 different alleles and 250 genomes. These mutations were excluded from Fig. 2 because of the scale difference.

Based on this analysis, we can estimate that the global prevalence of reported genomes showing PDC resistance mutations to the novel antipseudomonal cephalosporins in *P. aeruginosa* is 1.1% (1.8% if the V239A and R126H mutations are also included). Remarkably, we found that 42.5% of the genomes for which a resistance mutation was identified contained a new type PDC allele; therefore, the approach applied by the PDC analyzer web tool, which focuses on detecting specific AmpC mutations rather than detecting PDC allele variants, offers a major resolution in WGS-based resistance prediction to the new antipseudomonal cephalosporins.

### Concluding remarks

The implementation of whole-genome sequencing technologies has significantly contributed to our understanding of *P. aeruginosa* mutation-driven antibiotic resistance mechanisms. As *P. aeruginosa* antibiotic resistance genotypes should correlate with resistance phenotypes, a comprehensive and automated analysis of *P. aeruginosa* resistomes from whole-genome sequencing data would be of great benefit, for instance, for guiding therapeutic strategies and to develop new tools for antibiotic susceptibility prediction. In this work, we have introduced *Pa*REx, a pipeline specifically designed for this purpose, providing an automated and user-friendly solution that facilitates its implementation despite limited expertise on bioinformatics or in *P. aeruginosa* resistance mechanisms. In contrast to other software and databases developed for the detection of resistance markers in which a few resistance mutations are detected, *Pa*REx is able to analyze the presence of mutations in up to 221 chromosomal genes, including genes known to be involved in resistance to novel agents such as ceftolozane/tazobactam, ceftazidime/tazobactam, and/or cefiderocol. Moreover, in *Pa*REx, the detection of resistance mutations consists in identifying all variants and then filtering the natural polymorphisms. This is a potential advantage of *Pa*REx compared with other developed pipelines in which a list of mutations previously linked to antibiotic resistance is used for this purpose (23), thus avoiding the detection of novel resistance mutations and their use for other purposes such as genomic surveillance. Moreover, to our knowledge, the *Pa*REx pipeline is the only one including chromosomal genes linked to novel agents such as cefiderocol.

Although the *Pa*REx pipeline still has some margin for improvement, it represents a substantial advancement in the automated analysis of *P. aeruginosa* resistomes. In future versions, complementary bioinformatics approaches such as the analysis of resistance mutations directly on the *de novo* assemblies as well as the detection of gene absence and large genomic deletions will be integrated as it could be of some benefit for some particular scenarios and strains.

Along with *Pa*REx, we have developed a web tool for the detection of mutations in the *P. aeruginosa* chromosomal cephalosporinase AmpC, which have been demonstrated to compromise susceptibility to cefiderocol, ceftolozane/tazobactam, and/or ceftazidime/avibactam. This user-friendly tool represents a valuable resource for guiding therapies and for the monitoring of resistance to these last-resource antipseudomonal agents in settings with limited bioinformatic resources. Of note, a web tool for calculating the genotypic scores, and thus providing a genomic antibiogram, from the *Pa*REx final reports is currently under development.

## MATERIALS AND METHODS

### *The Pa*REx pipeline and databases

Instructions for setting up the *Pa*REx pipeline are available in the following repository: https://github.com/ARPBIGIDISBA/PaREx. As shown in Fig. 1, *Pa*REx comprises different steps detailed in the following subsections.

#### Read quality filtering

The first step of the *Pa*REx pipeline is quality filtering of raw Illumina paired-end reads using the open-source Java-based tool Trimmomatic v0.39 (24). By default, the following parameters are applied: LEADING:10, TRAILING:15, SLIDINGWINDOW:4:20, and MINLEN:100, parameters that aim to balance stringency with read retention, ensuring trimming of low-quality bases while preserving read length for *Pa*REx downstream analysis.

#### *De novo* genome assembly

Quality paired-end reads are then *de novo-*assembled using the St. Petersburg genome assembler algorithm SPAdes v3.15.5 (25). By default, *Pa*REx applies a multi-k-mer size strategy (-k 33, 55, 77, 99) to enhance assembly continuity and accuracy.

#### Multi-locus sequence typing

In the *Pa*REx pipeline, MLST is performed using the *de novo* genome assemblies with the mlst open-source software (https://github.com/tseemann/mlst), which uses the publicly available PubMLST scheme and database (https://pubmlst.org/). Both the Sequence Type and the MLST allelic profile (*acsA*, *aroE*, *guaA*, *nuoD*, *mutL*, *ppsA*, and *trpE*) are included in the *Pa*REx final reports along with the resistance genotype.

#### Acquired and mutational resistome profiling

As shown in Fig. 1, *de* n*ovo* assemblies are used for MLST and other additional downstream analyses, including the following: (1) detection of horizontally acquired antibiotic resistance genes (acquired resistome profiling), (2) PDC designation, and (3) evaluation of the structural integrity of the *P. aeruginosa* porin OprD.

Horizontally acquired antibiotic resistance genes are detected with the ResFinder analysis tool and its manually curated database (v.2.4.0) (9). The resulting ResFinder .json files are further processed with a Python-based script that removes *P. aeruginosa* intrinsic core resistance genes, incomplete ones, and/or duplicate results. In the final *Pa*REx reports, all acquired resistance genes along with their percentage of identity are listed and classified into four groups: beta-lactamases, aminoglycoside resistance genes contributing to tobramycin and/or amikacin resistance, fluoroquinolone resistance determinants, and other acquired resistance genes.

For PDC designation, *de novo* assemblies are processed with a Python-based script that uses the Basic Local Alignment Search Tool (BLAST+, v2.9.0-2) to compare assembled contigs against a custom-built database (https://arpbigidisba.com/pseudomonas-aeruginosa-derived-cephalosporinase-pdc-database/) in which all the PDC allele variants from the NCBI’s Pathogen Detection Reference Gene Catalog (last accession date: 03/06/2025) have been included. The detected PDC allele variant and any amino acid variations encountered relative to the PAO1 reference cephalosporinase sequence (PDC-1), which can be useful for understanding some resistance phenotypes, are included in the final reports.

Finally, to characterize the mutational resistome, *Pa*REx employs a reference-guided assembly approach. For the identification of single-nucleotide variants (SNVs) and insertions/deletions (InDels) between the *P. aeruginosa* PAO1 reference genome sequence (NC_002516.2) and isolates’ quality-filtered reads, *Pa*REx uses the Snippy tool applying default options (https://github.com/tseemann/snippy), and, then, the resulting snps.vcf file is parsed by a Python-based script.

In recent years, our understanding of mutation-driven antibiotic resistance mechanisms in *P. aeruginosa* has significantly advanced, primarily due to the screening of comprehensive mutant libraries and whole-genome sequencing data obtained from *in vitro* antibiotic resistance evolution assays, *in vivo* monitoring of antimicrobial resistance development, longitudinal analysis of sequential cystic fibrosis isolates, and from the characterization of epidemic high-risk clones. With this information, we have previously defined a panel of 164 chromosomal genes based on the PAO1 reference genome to analyze the mutational resistome of *P. aeruginosa* (15, 26) and for which we have recently defined the naturally occurring polymorphisms (20). For the *Pa*REx pipeline, we have extended this panel and included genes that have been linked to cefiderocol and/or ceftolozane/tazobactam and/or ceftazidime/avibactam resistance (27–31), as well as some additional genes related to the recycling and modification of *P. aeruginosa* peptidoglycans (Data S5).

From the snps.vcf file, *Pa*REx extracts this new panel that comprises a total of 221 chromosomal genes related to mutation-driven antibiotic resistance in *P. aeruginosa* and filters the naturally occurring polymorphisms from missense and nonsense mutations by using a custom-built database that includes about 2,875 natural polymorphisms (Data S5).

Finally, given the existence of different sequence variants of the gene encoding OprD (32) and its relevant role in carbapenem resistance, a specific module for assessing its structural integrity has been developed. In *Pa*REx, a custom Python-based script that uses the Basic Local Alignment Search Tool (BLAST+, v2.9.0-2) and a custom-built database including the OprD reference sequences of *P. aeruginosa* strains PAO1, LESB58, UCBP-PA14, MTB-1, FRD1, and F23197 is used for the evaluation of the OprD porin. This script first selects the most suitable reference sequence and then explores the integrity of the porin, thereby minimizing errors derived from the use of an inappropriate reference sequence.

#### PaREx final reports and outputs

As the final reports, *Pa*REx provides a csv file that integrates all generated results from all the isolates analyzed in the same batch along with individual summary reports for each of the isolates in pdf format. The csv file contains four different sheets corresponding to the basic and the extended resistomes, each provided in both raw and clean formats. The basic resistome includes the core set of chromosomic genes considered the main drivers of clinical resistance to classical antipseudomonal antibiotics, whereas the extended resistome includes additional genes that either have been associated with resistance to classical antipseudomonals but their effect is lower and/or has not been sufficiently demonstrated or are genes linked to resistance to newer β-lactam/β-lactamase inhibitor combinations (ceftolozane/tazobactam, ceftazidime/avibactam) and/or cefiderocol. Both clean resistome sheets contain only mutations with a documented or predicted impact on resistance as naturally occurring polymorphisms have been removed. In addition, during *Pa*REx execution, different output folders are generated containing intermediate files, such as the *de novo* assemblies or the output folder of Snippy, that are conserved and can be used by users for other purposes not covered in the pipeline.

#### PaREx running options

The *Pa*REx pipeline has been developed with flexibility in mind, allowing users to (1) skip some steps/modules, (2) adjust the running parameters for each step, and/or (3) run the analyses on single or multiple isolates. Detailed usage instructions are provided in the README file (https://github.com/ARPBIGIDISBA/PaREx).

#### PaREx lists and databases

##### Core resistance gene list

The manually curated ResFinder database includes genes that have been documented in the literature to be horizontally transferred among bacterial species, some of which are *P. aeruginosa* chromosomal genes that belong to its intrinsic core resistome (9). To identify the *P. aeruginosa* intrinsic core resistance genes included in the ResFinder database, we analyzed, along with PAO1 and PA14 reference genomes, the draft genomes from a collection of 461 *P. aeruginosa* clinical isolates that we have previously sequenced and which includes a set of 304 isolates from a multicenter study performed in 2017, which includes isolates exhibiting different antibiotic susceptibility profiles (13; European Nucleotide Archive Project Numbers: PRJEB40140 and PRJEB31047) and a set of 157 MDR and XDR isolates obtained from another multicenter study performed in 2022 (17; European Nucleotide Archive Project Number: PRJEB61879). This collection of 461 *P. aeruginosa* genomes was representative of all 17 Spanish regions and exhibited a high level of genetic diversity, with up to 192 different STs identified (Shannon diversity index, H’ = 4.47). All genes detected with ResFinder in at least 95% of the draft genomes were included in the *Pa*REx core resistance genes database. In addition, all detected blaOXA allele variants were individually investigated, and those classified as blaOXA-50 (PoxB) derivatives were also included in the database. Finally, and based on the current evidence, we excluded the *crpP* phosphotransferase as it does not appear to play any role in *P. aeruginosa* resistance to fluoroquinolones (33).

##### Natural polymorphisms database

For defining *P. aeruginosa* naturally occurring polymorphisms in the 221 chromosomal antibiotic resistance genes included in the mutational resistome analysis of the *Pa*REx pipeline, the same approach, consisting in the analysis of the genomes of 100 wild-type *P. aeruginosa* strains, that we used previously for defining the natural polymorphism in a subset of these genes was applied (13; European Nucleotide Archive project number PRJEB40140). Identified natural polymorphisms are listed in Data S5.

### Hardware and software setup

The *Pa*REx pipeline has been developed and executed on Ubuntu 20.04.6 LTS using Python v3.10.12 (packaged by conda-forge and compiled with GCC 12.3.0). Core third-party tools are installed using a dedicated Bash installer script that manages dependencies and sets up symbolic links for ease of use. Installation instructions are provided in the project’s GitHub repository and are compatible with any UNIX-based environment.

A system with at least 8 CPU cores, 16 GB RAM, and 512 GB SSD storage is recommended for optimal performance. The pipeline can also be executed on Windows systems using the Windows Subsystem for Linux 2 (WSL2).

### The PDC analyzer web tool

The PDC analyzer web tool is based on the Python-based script and custom-built database of the PDC designation module included in the *Pa*REx pipeline. For the list of AmpC mutations associated or that may contribute to resistance to the novel antipseudomonal cephalosporins, we performed a comprehensive and exhaustive literature review on PubMed using the following search terms: “ceftolozane tazobactam resistance OR ceftazidime avibactam resistance OR cefiderocol resistance AND pseudomonas AND mut*” (accession date: 29/05/2025).

### *P. aeruginosa* collections used for the validation of the functionalities of *Pa*REx and the PDC analyzer web tool

#### Analysis of a *P. aeruginosa* clinical isolate collection

For the validation of *Pa*REx and its functionalities, we sequenced and analyzed a set of 260 *P. aeruginosa* clinical isolates exhibiting different antibiotic susceptibility profiles (sequencing files have been deposited in the European Nucleotide Archive under study accession number PRJEB94805). For this purpose, four clinical isolates were randomly selected from each of the 66 hospitals (covering all 17 Spanish regions) participating in a national survey in 2022 (17). Minimum inhibitory concentrations of piperacillin/tazobactam (4/4–256/4 mg/L), ceftazidime (1–64 mg/L), cefepime (1–64 mg/L), ceftolozane/tazobactam (0.5/4–32/4 mg/L), ceftazidime/avibactam (0.5/4–32/4 mg/L), aztreonam (2–128 mg/L), imipenem (0.5–64 mg/L), meropenem (0.5–64 mg/L), ciprofloxacin (0.12–16 mg/L), tobramycin (0.25–32 mg/L), amikacin (2–128 mg/L), and colistin (0.5–16 mg/L) have been previously determined by broth microdilution using SensiTitr panels (Plate Code:FRCNRP2, Thermo Fisher Diagnostics, S.LU) and using EUCAST 2025 (v15.0), and clinical breakpoints were used for interpretation of SIR categories. The modR profile was defined as resistance to at least 1 agent in 1 or 2 of 7 antibiotic classes including antipseudomonal penicillins + β-lactamase inhibitor combinations (piperacillin/tazobactam), antipseudomonal cephalosporins (ceftazidime and cefepime), monobactams (aztreonam), antipseudomonal carbapenems (imipenem and meropenem), fluoroquinolones (ciprofloxacin), aminoglycosides (tobramycin and amikacin), and polymyxins (colistin); the MDR profile is defined as resistance to at least 1 agent in at least 3 of 7 antibiotic classes, and the XDR profile is defined as resistance to at least 1 agent in all but 1 or 2 antibiotic classes (34).

#### Analysis of the NCBI Microbial Browser for Identification of Genetic and Genomic Elements (MicroBIGG-E) database

Recently, Mack and collaborators (22) explored the presence of β-lactamase alleles in more than 30,000 *P. aeruginosa* isolates collected worldwide and deposited in the NCBI Pathogen Detection databases to gain insights into the *P. aeruginosa* β-lactamases. In this work, we used the PDC analyzer web tool to analyze this global collection and to determine the global prevalence of mutations linked to resistance to the novel antipseudomonal combinations ceftolozane/tazobactam, ceftazidime/avibactam, and/or cefiderocol.

### *Pa*REx availability and requirements

Project name: ***P****seudomonas* ***a****eruginosa* **R**esistome **Ex**plorer

Project home page: https://github.com/ARPBIGIDISBA/PaREx.

Operating systems: Unix (Ubuntu 20.04.6 LTS) and Windows (Windows Subsystem for Linux 2)

Programming language: Python

Other requirements: Python 3.8.10 or higher

License: Creative Commons Attribution-NonCommercial-ShareAlike 4.0 International (CC BY-NC-SA 4.0)

Any restrictions to use by non-academics: Commercial use is prohibited without prior permission. Attribution and the same license are required for modifications.

## SUPPLEMENTAL MATERIAL

Data S1 aac.01326-25-s0001.xlsx Compiled results for the 261 isolates used for PaREx validation.

Data S2 aac.01326-25-s0002.pdf Examples of the individual pdf final summary reports generated by PaREx.

Data S3 aac.01326-25-s0003.xlsx MIC values and genotypic scores obtained for the 260 isolates used for PaREx validation.

Data S4 aac.01326-25-s0004.pdf Examples of different results obtained from the PDC analyzer web tool.

Data S5 aac.01326-25-s0005.xlsx Panel of 221 chromosomal genes comprising the mutational resistome of *P. aeruginosa*.

ASM does not own the copyrights to Supplemental Material that may be linked to, or accessed through, an article. The authors have granted ASM a non-exclusive, world-wide license to publish the Supplemental Material files. Please contact the corresponding author directly for reuse.
