# Supplementary material for: PaREx: an open-source pipeline for the automated analysis of Pseudomonas aeruginosa resistomes from whole-genome sequences
Source: Antimicrob Agents Chemother. 2026 Mar 16;70(4):e01326-25. doi: 10.1128/aac.01326-25 (PMC13041343; doi:10.1128/aac.01326-25)
Supplement: Data S2 — Examples of the individual pdf final summary reports generated by PaREx. [file aac.01326-25-s0002.pdf]

Supplementary material 2: Examples of the individual pdf summary reports generated by *Pa*REx.

Isolate ID: CAT09-004_46

**Sequence Type**	
**ST**	111
**MLST allelic profile**	acsA(17) aroE(5) guaA(5) mutL(4) nuoD(4) ppsA(4) trpE(3)
**PDC**	
**Aminoacid substitutions (vs PDC-1)**	T105A
**PDC variant (RefSeq protein ID)**	PDC-3 (WP_003121934.1)
**Horizontally acquired resistome**	
**Beta-lactamases**	blaOXA-46 (100.00%)
**Aminoglycosides resistance genes**	aac(6')-Ib3 (99.82%)
**Quinolones resistance genes**	
**Other resistance genes**	sul1 (100.00%)
**Mutational resistome**	
ampD (V10G), *gyrA* (T83I,V671I,G860S,D893E,A900G,S903A), *mexT* (nt240_247del), *mexY* (G530S), *nalD* (nt398_399del), *parC* (S87L), *parS* (L137P,R279Q), *PBP1b* (M715K), *ponA* (A615_D616insP)	

Isolate ID: CLM02-001

**Sequence Type**	
**ST**	175
**MLST allelic profile**	acsA(28) aroE(22) guaA(5) mutL(3) nuoD(3) ppsA(14) trpE(19)
**PDC**	
**Aminoacid substitutions (vs PDC-1)**	
**PDC variant (RefSeq protein ID)**	PDC-1 (WP_003101289.1)
**Horizontally acquired resistome**	
**Beta-lactamases**	blaOXA-2 (100.00%), blaVIM-20 (100.00%)
**Aminoglycosides resistance genes**	aph(3')-VI (100.00%), ant(2'')-Ia (100.00%)
**Quinolones resistance genes**	
**Other resistance genes**	
**Mutational resistome**	
armZ (V266M), *gyrA* (T83I,D87N), *mexT* (nt240_247del), *mexZ* (G195E), *oprD* (nt49ins5 [LESB58]), *parC* (S87W,L168Q), *ponA* (E245D,A615_D616insP)	
