# Supplementary material for: PaREx: an open-source pipeline for the automated analysis of Pseudomonas aeruginosa resistomes from whole-genome sequences
Source: Antimicrob Agents Chemother. 2026 Mar 16;70(4):e01326-25. doi: 10.1128/aac.01326-25 (PMC13041343; doi:10.1128/aac.01326-25)

Supplementary material 4: Examples of the results generated by the PDC analyzer web tool.

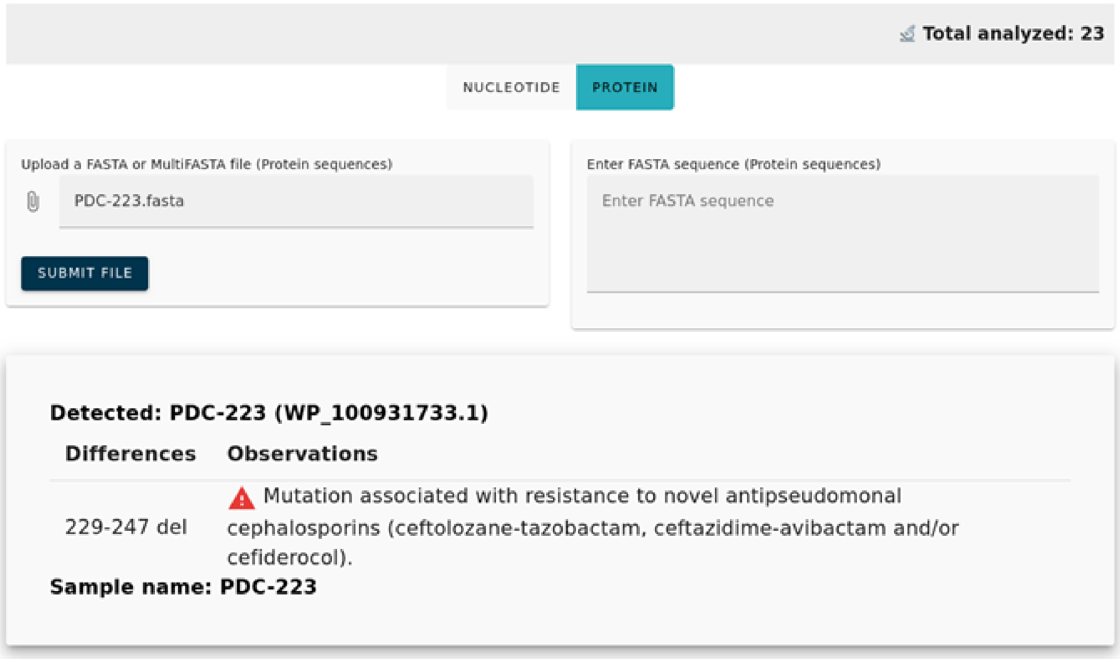

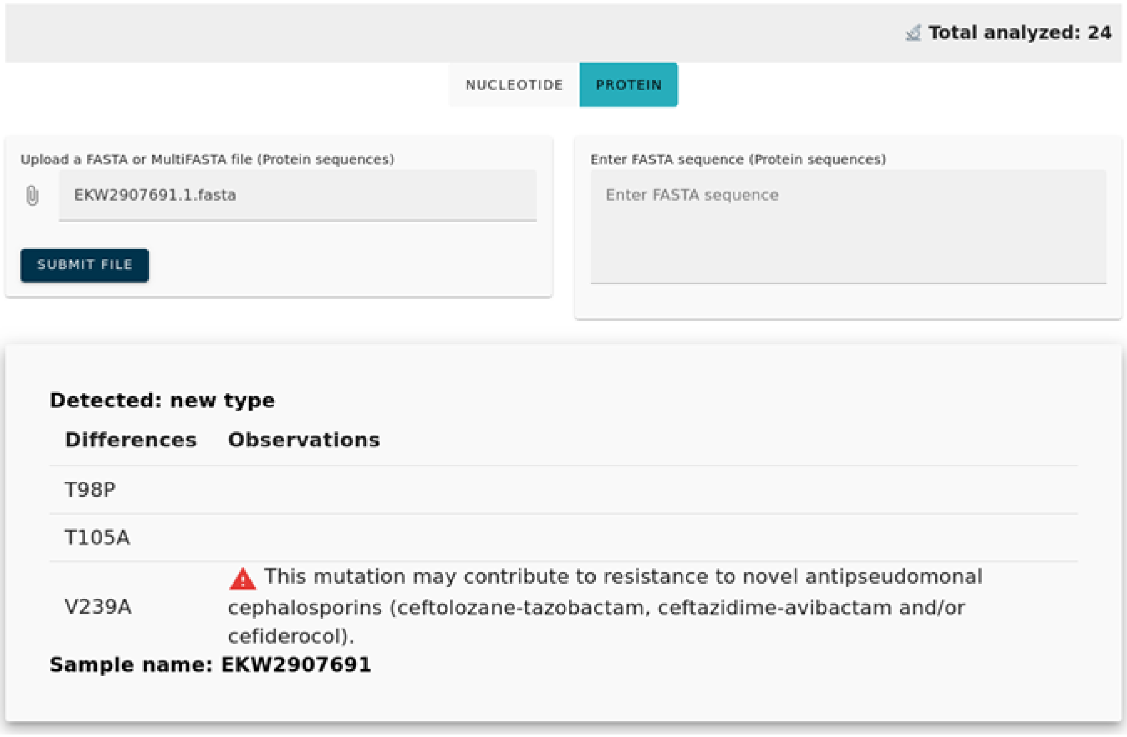

Supplement: Data S4 — Examples of different results obtained from the PDC analyzer web tool. [file aac.01326-25-s0004.pdf]
